# Structural basis of DUX4/IGH-driven transactivation

**DOI:** 10.1038/s41375-018-0093-1

**Published:** 2018-03-15

**Authors:** Xue Dong, Weina Zhang, Haiyan Wu, Jinyan Huang, Ming Zhang, Pengran Wang, Hao Zhang, Zhu Chen, Sai-Juan Chen, Guoyu Meng

**Affiliations:** 1 State Key Laboratory of Medical Genomics, Shanghai Institute of Hematology, Rui-Jin Hospital, Shanghai JiaoTong University School of Medicine and School of Life Sciences and Biotechnology, Shanghai JiaoTong University, 197 Ruijin Er Road, Shanghai, 200025 China; 2 Key Laboratory of Systems Biomedicine, Shanghai Center for Systems Biomedicine, Shanghai JiaoTong University, 800 Dong Chuan Road, Shanghai, 200240 China

## Abstract

Oncogenic fusions are major drivers in leukemogenesis and may serve as potent targets for treatment. DUX4/IGHs have been shown to trigger the abnormal expression of ERG_alt_ through binding to DUX4-Responsive-Element (DRE), which leads to B-cell differentiation arrest and a full-fledged B-ALL. Here, we determined the crystal structures of *Apo*- and DNA_DRE_-bound DUX4_HD2_ and revealed a clamp-like transactivation mechanism via the double homeobox domain. Biophysical characterization showed that mutations in the interacting interfaces significantly impaired the DNA binding affinity of DUX4 homeobox. These mutations, when introduced into DUX4/IGH, abrogated its transactivation activity in Reh cells. More importantly, the structure-based mutants significantly impaired the inhibitory effects of DUX4/IGH upon B-cell differentiation in mouse progenitor cells. All these results help to define a key DUX4/IGH-DRE recognition/step in B-ALL.

## Introduction

Oncogenic fusions are important causes/targets in leukemia and related treatment [1, 2]. Acute lymphoblastic leukemia (ALL) is the most common childhood malignancy. B-ALL, which stems from the clonal proliferation of B lineage progenitors [3, 4], constitutes 80% of ALL cases [5]. Very recently, using next-generation sequencing technologies, a novel B-ALL oncogenic driver *DUX4/IGH*, derived from the insertion of chromosome fragments containing *DUX4* gene into the *IGH* locus, has been reported in ~7% of B-ALL patients [3, 6, 7].

Double homeobox 4 (DUX4) is the core component of human subtelomeric macrostalelite D4Z4 [8]. It has been shown that 11 to >150 D4Z4 repeats can be identified in chromosome 4 and 10. In each of the D4Z4 repeat (~3.3 kb), it constitutes a single copy of the intronless *DUX4* gene. At physiological condition, DUX4 is usually silenced [9]. In pathogenic condition known as facioscapulohumeral muscular dystrophy (FSHD) [10], the D4Z4 repeats are contracted to 1–10. The change of chromatin packing is thought to be a critical factor that leads to the abnormal expression of DUX4. Consistently, in B-cell ALL, the abnormal expression of DUX4/IGHs derived from chromosome translocation is also demonstrated to be a driver factor of leukemogenesis [3]. Furthermore, the deregulation of DUX4 is frequently associated with the biogenesis of ERG_alt_ [4]. Very briefly, ERG is an ETS-family transcription factor playing important role in normal hematopoietic regulation [11] and is involved in the pathogenesis of T-ALL and acute myeloid leukemia [12–14]. ERG_alt_, derived from an abnormal transcription starting from the intron 6 of *ERG*, could induce lymphoid leukemia in *Arf*^−/−^ mice [4]. Therefore, the ERG_alt_ biogenesis is recognized as a reporter and a potent secondary hit in DUX4-driven leukemogenesis. However, due to the lack of structural information, it is not clear how DUX4/IGH recognizes, binds, and activates the transcription of its target genes via the double homeobox (HD1–HD2) domain.

In this report, we determined the crystal structures of *Apo*-DUX4_HD2_ and DNA-bound complex. The atomic structures, together with functional characterization, reveal a two-step clamp-like binding mechanism between double homeobox and TAATCTAAT DNA site, the latter of which is frequently observed in DUX4 target genes, and hence termed DUX4-Responsive-Element (DRE). Consistent with previous observation that a single HD domain is not sufficient for productive protein–DNA interaction, our data showed that both HD1 and HD2 are essential in DRE recognition. Single mutation in the major-groove-binding interfaces of HD1 and HD2 can significantly impair DUX4-DRE binding and DUX4-driven transactivation. When investigated in the context of DUX4/IGH and B-cell differentiation, structure-based mutations also abolish DUX4/IGH’s transcriptional activities and their aggressive/inhibitory roles upon B-cell differentiation. All these results have led to the novel proposal that the DUX4-DRE recognition and two-step clamp-like binding mechanism are critical for DUX4/IGH-driven transactivation and B-ALL leukemogenesis.

## Materials and methods

Information concerning bacterial and eukaryotic cells, DNAs, and other materials used in this study is described in Supplementary Data. The experimental details of structure determination, functional/genetic characterizations including biolayer interferometry assay, transactivation assay, luciferase assay, B-cell differentiation assay, RNA-seq analysis etc. are also described in Supplementary Data. The statistics of the data collection and structure refinement are shown in Supplementary Table 1. Coordinates of *Apo*-DUX4_HD2_ and DUX4_HD2_-DNA_DRE_ have been deposited into the Protein Database Bank. The PDB codes are 5Z2S and 5Z2T, respectively.

## Results

### DUX4/IGH-driven transactivation in B-ALL

**Fig. 1:**
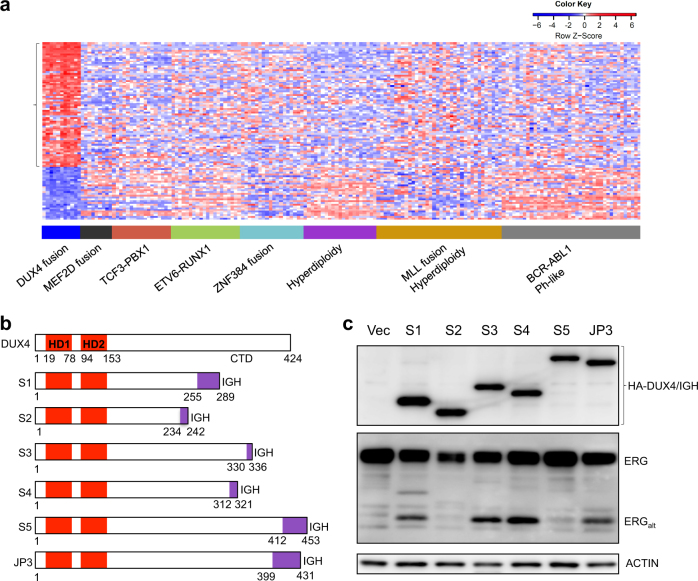
DUX4/IGH-driven transactivation in Chinese B-ALL patients. **a** Heatmap of abnormal gene expression in DUX4-ALL patients (blue) and other B-ALL subgroups including MEF2D fusion (black), TCF3-PBX1 (red), ETV6-RUNX1 (green), ZNF384 (cyan) fusion, hyperdiploidy (purple), MLL fusion (brown), and BCR-ABL1 (gray). The DUX4-driven transactivation of genes in patient samples is highlighted with bracket symbol on the left. **b** Domain arrangement of WT DUX4 and its oncogenic fusions obtained from Chinese patients S1 to S5. DUX4/IGH_JP3_ was previously demonstrated to induce leukemia in mice [3]. **c** Transactivation activity of DUX4/IGHs. Western blot analysis of ERG_alt_ in Reh cells overexpressing HA-tagged DUX4/IGH fusions. The bracket is used to highlight the expression of DUX4/IGHs

We have previously reported the genomic profiling of adult and pediatric B-cell ALL based on a cohort of Chinese patients [6]. Using RNA-seq technology, we observed that the abnormal expression of DUX4/IGHs often led to the overexpression of a subset of DUX4 target genes including *PCDH17*, *STAP1*, *AGAP1, ERG*_*alt*_, etc. (Fig. 1a). The expressions of DUX4 fusions were often associated with the genetic events of *NRAS* mutations (Supplementary Table 2). The breakpoints in DUX4 fusions were all located in the C-terminus of the protein (Fig. 1b). Consistent with previous observations [3, 4], human leukemia cell line Reh cells harboring the newly identified DUX4/IGHs showed the expression of ERG_alt_ (Fig. 1c). All the DUX4 truncated mutants derived from Chinese patients harbor an intact N-terminal HD1–HD2 domain. This is also consistently observed in other B-ALL patients reported by the Japanese and American investigators [3, 4], suggesting that the double homeobox HD1–HD2 is critical for DUX4/IGH activity and B-ALL leukemogenesis. Supportively, it has been reported that wild-type (WT) DUX4 can selectively bind to DNA [4, 15–17]. Using published ChIP-seq data [10], we have investigated a number of DUX4/IGH target genes including ERG_alt_ site TAATCTCAT (Supplementary Figure 1a,b). This has led to the discovery of a DUX4/IGH consensus binding site TAATCTAAT, and hence termed DRE (Supplementary Figure 1c).

### Crystal structures of Apo- and DNA_DRE_-bound DUX4_HD2_

**Fig. 2:**
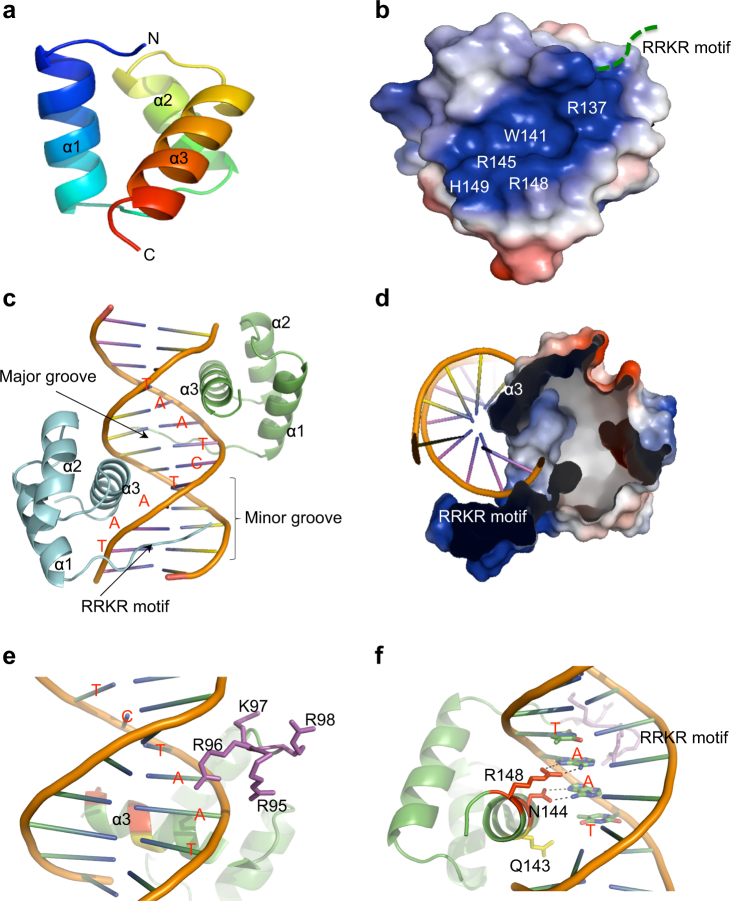
Crystal structures of *Apo*- and DNA-bound DUX4_HD2_. **a** Apo-form of HD2 domain. The homeobox subdomain is shown in cartoon representation. The N/C-termini and α1/α2/α3 are labeled. **b** Electrostatic surface of *Apo*-DUX4_HD2_. The highly positively charged patch in *Apo*-HD2 is delineated by residues Arg137, Trp141, Arg145, Arg148, and His149. The flexible N-terminal poly-Arg motif is shown with a dashed line. **c** Crystal structure of DUX4_HD2_-DNA_DRE_ complex. The HD2 molecules and TAATCTAAT sequences (DNA_DRE_) are shown in cartoon representation and colored in cyan, green, and orange, respectively. **d** The electrostatic surfaces of the N-terminal loop_RRKR_ and C-terminal α3 helix that engage with DNA binding. **e** The interface between the N-terminal RRKR motif and DNA_DRE_ minor groove. The poly-Arg residues are shown in stick representation. **f** The interface between the α3 helix and DNA_DRE_ major groove. Gln143 (yellow), Asn144 (red), and Arg148 (red) are shown in stick representation. The hydrogen bonds are highlighted with dashed lines. The TAAT nucleotides are labeled

In order to investigate how DUX4/DUX4 fusions recognize DNA, we have determined the crystal structures of *Apo*-DUX4_HD2_ and DNA_DRE_-bound complex at the resolutions of 1.5 and 2.6 Å, respectively (Fig. 2). The *Apo* structure is a classic homeobox fold with three α-helices, termed α1, α2, α3, respectively (Fig. 2a). The N-terminal α1, α2 helices are anti-parallel. The α3 helix is perpendicular to the axis of α1/α2 helices. Compatible with its DNA binding activity, the electrostatic surface of *Apo*-DUX4_HD2_ reveals a highly positively charged pocket delineated by residues Arg137, Trp141, Arg145, Arg148, and His149 in the α3 helix (Fig. 2b).

The HD2-DNA complex structure reveals a clamp-like DNA-binding mechanism, in which the N-terminal R_95_RKR_98_ loop and the α3 helix interact with the TAAT site in the minor and major grooves, respectively (Fig. 2c, d). In the minor groove, the N-terminal R_95_RKR_98_ loop adopts an extended configuration making loosen interaction with the DNA backbone (Fig. 2e). In the asymmetry unit (ASU), two DUX4_HD2_ molecules were observed. The N-terminal R_95_RKR_98_ loop is highly flexible and might adopt different configurations for the binding of DNA minor groove. The two HD2 molecules in ASU are superimposed in the context of DNA binding. Upon DNA binding, Arg96/Lys97 or Arg95/Lys97 are utilized to make direct contract with the phosphate groups of DNA backbone in the minor groove (Supplementary Figure 2a). In compatible with the negatively charged nature of DNA molecules, the highly positively charged N-terminal RRKR loop could serve as important recruitment platform to initiate DNA binding via electrostatic interaction (see structure-based characterization below).

In the major groove, the α3 helix forms direct hydrogen bonds with DNA (Fig. 2f). The residues Arg137, Trp141, Arg145, and His149 are interacting with the DNA backbone (Supplementary Figure 2b). Compared with the *Apo*-DUX4_HD2_ structure, the guanidinium head group of Arg137 in the DNA-bound complex shifts 2.9 Å upward to form two direct hydrogen bonds with the phosphate groups in the DNA backbone. In parallel, His149, located in the opposite end of the α3 helix, moves 1.7 Å upward, flanking Arg145 via a side-chain π–π interaction. As a result, the side chains of Trp141, Arg145, and His149 all adopt extended configurations, with the alkyl side-chain in parallel to each other and the charged head groups pointing towards DNA. This has given rise to a pedestal-like base, on which the DNA backbone can rest. Inside the DNA major groove lie the residues Gln143, Asn144, and Arg148, termed QNR motif (Fig. 2f). The side-chains of these residues insert deep into the major groove reading the “barcode” of T_1_A_2_A_3_T_4_. Gln143 is located 4.6 Å away from the DRE nucleotides. Structural superimposition of *Apo-* and DNA-bound DUX4_HD2_ reveals interesting sidechain reshuffles of Asn144 and Arg148 upon the recognition of DRE. The carboxamide head group of Asn144 rotates 30° anti-clockwise to make two direct hydrogen bonds with the A_2_ nucleotide. In a concerted fashion, the guanidinium head group of Arg148 swings 5.3 Å into the major groove to form hydrogen bonds with the A_3_ site. The minor- and major-groove binding residues including RRRR_HD1_–RRKR_HD2_, QNR_HD1_–QNR_HD2_ motifs are highly conserved in DUX protein family (Supplementary Figure 2c), among which HD1 shares 54% sequence identity with HD2. In particular, Asn144 is strictly conserved in homeobox superfamily (Supplementary Figure 2d), highlighting the significance of DNA binding ability in DUX4/IGH-driven transactivation.

### DUX4-DRE is important for DNA binding, transactivation, and B-cell differentiation

**Fig. 3:**
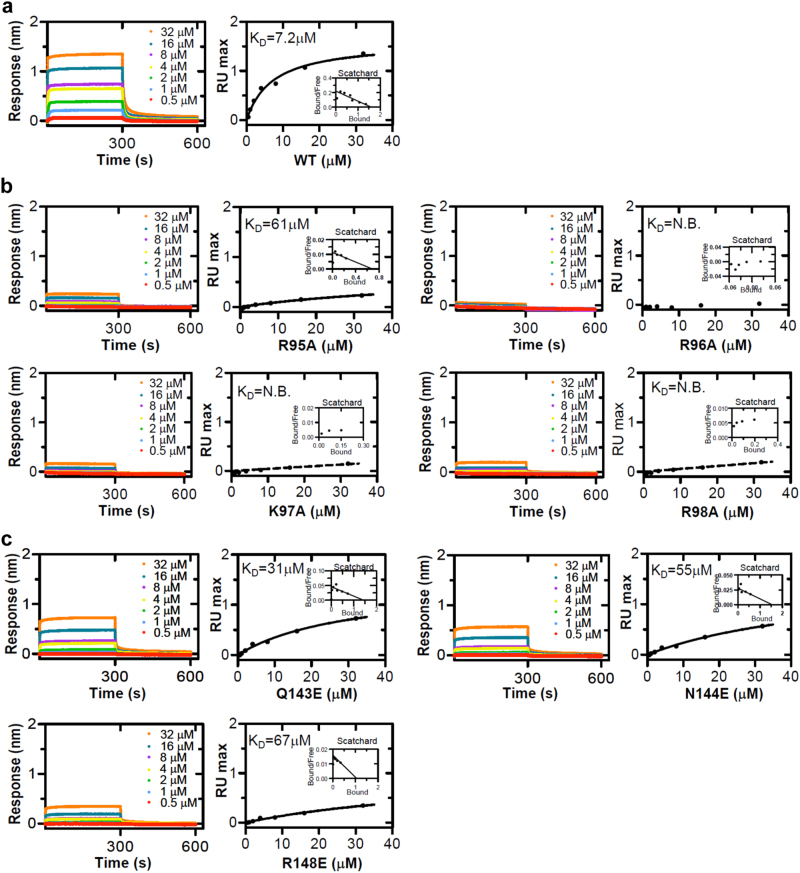
The BLI characterization of DNA_DRE_ binding by DUX4_HD2_ and mutants. **a** The interaction between DUX4_HD2_ and DNA_DRE_. **a** WT DUX4_HD2_. **b** DUX4_HD2_ mutations in the minor groove binding residues, i.e., poly-Arg motif. **c** DUX4_HD2_ mutations in the major groove binding residues, i.e., QNR motif. A gradient concentration of 0.5–32 μM recombinant DUX4_HD2_ was used. Under these experiment conditions, the DNA_DRE_ was immobilized on the probe. Left, the association and dissociation curves. Right, the binding curves and *K*_*D*_ values derived from Scatchard plots [32]

To characterize the crystal structure of DUX4_HD2_-DNA_DRE_, the biolayer interferometry (BLI) was used. The recombinant DUX4_HD2_ and DUX4_HD1–HD2_ were shown to bind to a DRE sequence at *K*_*D*_ values of 7.2 and 1.4 μM, respectively (Fig. 3a and Supplementary Figure 3a). By contrast, the mutations of R95A, R96A, K97A, and R98A in the poly-Arg motif significantly abolished the DNA binding activity of DUX4_HD2_ (Fig. 3b). Consistently, in the α3 helix region, Q143E, N144E, and R148E resulted in 4- to 9-fold weaker binding. The measured *K*_*D*_ values of these mutants were 31, 55, and 67 μM, respectively (Fig. 3c). Supportively, similar structure-based mutations in DUX4_HD1–HD2_ completely abrogated DNA binding activity (Supplementary Figure 3b).

**Fig. 4:**
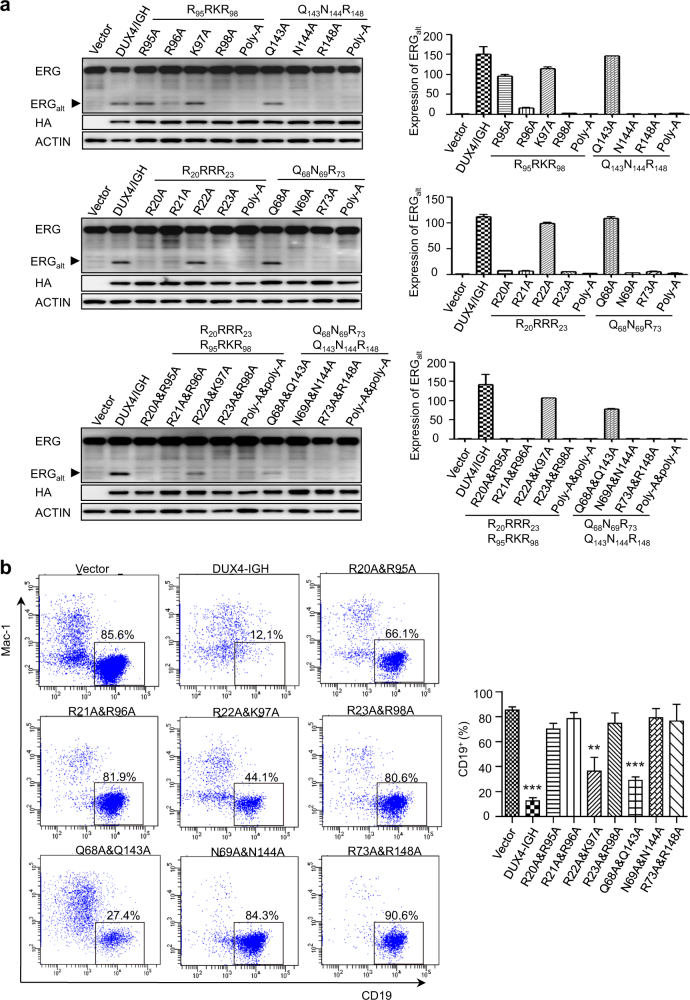
DRE recognition is important for DUX4/IGH-driven transactivation and B-cell differentiation. **a** Transactivation activity of DUX4/IGH and mutants in Reh cells. Left column, the biogenesis of ERG_alt_ in Reh cells that contain WT and mutant DUX4/IGHs was detected by western blot. Right column, the real-time PCR technique was used to confirm the DUX4-driven transactivation at mRNA level. Top row, mutations in HD2. Middle row, mutations in HD1. Bottom row, double mutations in HD1 and HD2. All experiments have been done at least with three independent replicates. **b** *In vitro* B cell differentiation assay of DUX4/IGH and mutants. FACS technique was used to analyze the effect of DUX4/IGH and mutants on the differentiating ability of mouse progenitor cells (Lin^−^/c-Kit^Low^) into CD19 positive B cells. All experiments have been done at least with three independent replicates. Values are means ± SE

As previously reported [4], the ERG_alt_ biogenesis could be used to monitor the transactivation activities of DUX4/IGH and mutants. The WT DUX4/IGH transactivated the expression of ERG_alt_ (Fig. 4a and Supplementary Figure 4). Contrarily, the perturbation of DNA binding residues abrogated the biogenesis of ERG_alt_. The poly-Ala mutations of RRRR_HD1_–RRKR_HD2_ and QNR_HD1_–QNR_HD2_ motifs in either HD1 or HD2 completely abolished the transactivation activity of DUX4/IGH, suggesting that HD1 and HD2 domains are both required for DRE recognition.

In order to investigate which DNA-binding motif (RRRR_HD1_–RRKR_HD2_ or QNR_HD1_–QNR_HD2_) could be more important, a series of single mutations were designed. The DUX4/IGH-driven transactivation activities (i.e., the abnormal biogenesis of ERG_alt_) of wild type and mutants were monitored in Reh cells. The single mutation in QNR motifs (i.e., N69A, R73A in HD1 and N144A, R148A in HD2) was sufficient to inhibit the DNA_DRE_ recognition and abolish subsequent ERG_alt_ transactivation (Fig. 4a). Interestingly, the single mutations, Q68A and Q143A, failed to inhibit DUX4/IGH-driven transactivation. In marked contrast, the double Q68/143A mutation dramatically reduced ERG_alt_ biogenesis, suggestive of rescue effect between HD1 and HD2 (Fig. 4a). Consistently, the complementary binding mode was also observed in the single mutations of RRRR_HD1_–RRKR_HD2_. Although single mutation in RRKR_HD2_ could preclude the formation of DUX4_HD2_-DNA_DRE_ (Fig. 3b), most single mutations in RRKR_HD2_ were less destructive, resulting in partial disruption of DUX4-driven transactivation (Fig. 4a), implying the DNA binding between DUX4/IGH and DNA might be rescued by the equivalent poly-Arg motif in HD1. Indeed, when an extra Arg/Lys to Ala mutation was engineered in the equivalent position (such as R20/95A, R21/96A, R22/K97A, R23/98A), the accumulative impacts were very clear in spite of the fact that the QNR_HD1_–QNR_HD2_ motif remained intact in these mutants (Fig. 4a). Supportively, similar results can be obtained using NALM6 cells (Supplementary Figure 4). All these results support a concerted binding clamp-like binding between HD1 and HD2. Furthermore, as shown in Fig. 4a, the single mutations in HD1 appear to be more destructive than those of HD2, prompting the hypothesis that two HDs might have different roles in DNA binding, in which HD1 might be more affinitive subunit to engage DRE site.

Next, we want to know whether the DRE recognition via HD domains could play a key role in disease mechanism. As demonstrated elsewhere [3], the leukemogenic activity of DUX4/IGH is constantly associated with the arrest of B-cell differentiation. We therefore addressed the phenotypic effect of distinct fusion proteins with WT or mutated DRE-binding motifs. In control experiment, ~85% primary murine progenitors transfected with vehicle (empty vector) could undergo normal B-cell differentiation as assessed by flow cytometry using antibodies against mouse CD19 (Fig. 4b). DUX4/IGH-transfected cells, by contrast, showed much less B-cell differentiation (~12%), in concordance with its transforming ability and leukemogenic effects [3]. The perturbations in the RRRR_HD1_–RRKR_HD2_ and QNR_HD1_–QNR_HD2_, which hampered the DNA binding and transactivation, also abrogated the inhibitory activity of DUX4/IGH on B-cell differentiation. As shown in Fig. 4b, most mutations rescued the lymphoid lineage differentiation at levels similar to that of the cells transfected with vehicle (i.e., 66 – 90% differentiation for R20/95A, R21/96A, R23/98A, N69/144A, R73/148A). Of note, cells transfected with R22/K97A and Q68/143A still bore certain levels of ERG_alt_ expression (Fig. 4a) and showed only partially restored differentiation compartments in FACS analysis (44 and 27%, respectively) (Fig. 4b). These results taken together not only support our observation in the crystal structure of DUX4_HD2_-DNA_DRE_, but also suggest DUX4_HD1–HD2_-DRE interacting motif as a potential therapeutic target for future treatment against B-ALL harboring DUX4/IGH fusion. Supportively, the structure-based mutations also inhibit the apoptotic function of WT DUX4 (Supplementary Figure 5a), and its transactivation ability on target gene as monitored by luciferase assay (Supplementary Figure 5b). Altogether, these have led to hypothesis that WT DUX4 and DUX4/IGH might share a similar DNA binding mechanism (see below).

**Fig. 5:**
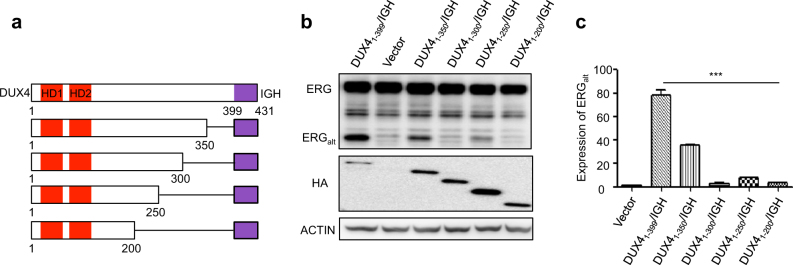
The effect of C-terminal domain in DUX4/IGH-driven transactivation. **a** Scheme of CTD truncated mutants. The biogenesis of ERG_alt_ in Reh cells by WT DUX4/IGH and C-terminal truncated mutants were monitored by western blot (**b**) and RT-PCR (**c**)

However, until now, it is not clear how the C-terminal domain (CTD) of DUX4, whose molecular boundary is not yet clearly defined, might contribute to the transactivation activities of WT DUX4 and DUX4/IGHs. WT DUX4, overexpression of which often triggers apoptosis, is the main reason of FSHD disease [10]. In marked contrast, the loss/break-down of CTD has enabled DUX4/IGH to cause B-cell arrest and proliferation, and hence serves as an oncogenic driver in leukemia [3, 4]. In order to understand how CTD might influence the transactivation activity of DUX/IGH, a series of CTD truncated mutants were designed (Fig. 5a). Interestingly, when the CTD was restricted to residue 200, the DUX4/IGH activity was significantly impaired (Fig. 5b, c). Consistently, this correlates with the observation in B-ALL patients. The shortest version of DUX4/IGH derived from Patient S2 also displayed less ERG_alt_ expression (Fig. 1c), suggestive of CTD contribution in DUX4/IGH and B-ALL. However, the size of CTD is not the only structural factor in DUX4/IGH’s transactivation activity. DUX4_1-412_/IGH from Patient S5 displayed less ERG_alt_ expression than those shorter fusion derivatives from Patients S3 and S4 (Fig. 1b, c). Similar results could be observed in CTD truncated mutants (Fig. 5b, c), suggesting that the overall structure/fold of CTD, which remains elusive, might play critical role in DUX4/IGH-driven transactivation.

### Abnormal transactivation controlled by DUX4_HD1–HD2_-DNA_DRE_

**Fig. 6:**
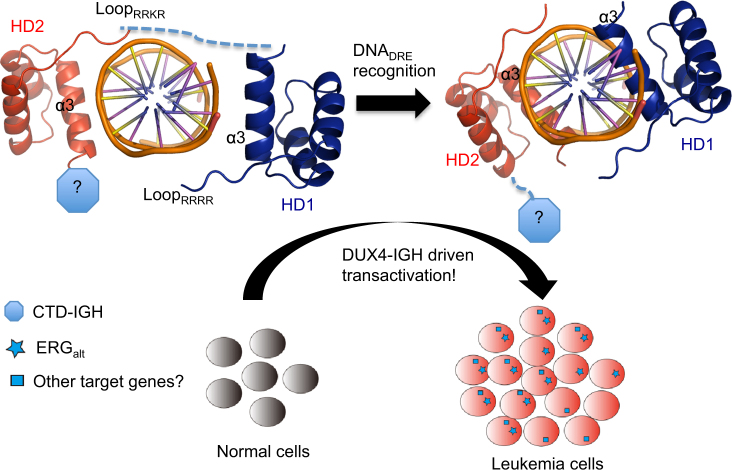
Oncogenic network controlled by DUX4_HD1–HD2_-DNA_DRE_ recognition in B-ALL pathogenesis. A two-step cooperative binding/transactivation mechanism via HD1 and HD2 domains. In the first step (top-left panel), poly-Arg motifs lead the initial DNA binding via a non-specific eletrostatic attraction. This in turn primes the second step (top-right panel) for the intimate/specific clamp-like association between DUX4-DNA_DRE_ via QNR_HD1_–QNR_HD2_ motifs. The two-step DUX4/IGH is a critical step in B-ALL leukemogenesis (low panel). Based on 54% sequence identity, the HD1 model in this figure is built by homology modeling using DUX4_HD2_ coordinates. The covalent linkage between HD1 and HD2 is shown in dashed line. CTD is indicated with octagon. The symbols “?” are used to highlight/prompt further investigations to understand (1) what role CTD might play in DUX4- and DUX4/IGH-driven transactivations; (2) whether DUX4/IGH target genes such as *AGAP1* could accelerate/or is required in DUX4/IGH-mediated leukemogenesis

The RNA-seq data mining of our 172 patients showed that the expression profile of DUX4/IGH positive ALL was significantly different from other B-ALL subtypes (Fig. 1a). Notably, a group of genes such as *AGAP1*, *CHST2*, *CLEC12A*, *PCDH17*, *PTPRM*, *STAP1*, etc., which all contain DREs, were found upregulated in patients harboring DUX4 fusions. This is further supported by the observation that knock-down (KD) of DUX4/IGH through shRNA in NALM6 cells [3], which contains an endogenous DUX4/IGH fusion, downregulated these target genes (Supplementary Figure 6a). We next investigated the function of these DUX4/IGH-targeted genes by FACS profiling and apoptotic assay. Firstly, FACS profiling showed that the over-expression of *AGAP1*, but none of other target genes, led to a partial arrest of B-cell differentiation in murine progenitor cells (Supplementary Figure 6b). Secondly, the genetic KD of *AGAP1* in NALM6 cells exerted a significant impact on apoptosis, whereas KD of other DUX4/IGH target genes (except for *CHST2*) did not show obvious apoptotic effects (Supplementary Figure 6c). More importantly, as monitored by luciferase assays, the structure-based mutations significantly abolished the DUX4/IGH-driven transactivation of *AGAP1* (Supplementary Figure 6d). Supportively, abnormal expression of *AGAP1* is frequently observed in B-ALL patients with DUX4 fusions reported elsewhere [4, 18]. These results highlight a critical DUX4_HD1–HD2_-DNA_DRE_ recognition in B-ALL pathogenesis (Fig. 6). However, although *AGAP1* displays potential in B-cell differentiation, further investigation such as *in vivo* characterization involving the use of knock-out mice model is required to establish the leukemogenic role of DUX4/IGH target gene.

## Discussion

### Protein diversity in DUX4

DUX4, a member of homeobox protein family, is exclusively found in placental mammals [19, 20]. Compared to other homeobox proteins, DUX4 contains two adjacent HD1 and HD2 domain, connected by a short linker of 15 residues. Thus far, no structural information is available for the double homeobox. In this section, we will focus our discussion on the protein diversity in DUX4 double homeobox.

It has been proposed that DUX4 belongs to the PAIRED (PRD) homeobox subclass because their HD domains share the most sequence identity (Supplementary Figure 2d) [21]. In addition, the tandem arrangement of double homeobox is in striking similarity with the domain architecture observed in PRD proteins. The PAI sub-domain is a homeobox, and the RED sub-domain can fold into a HD-like structure with three α-helices [20, 22]. Hence PAI-RED appears to be a double-homeobox-like structure. However, it has been shown that the RED sub-domain is not really a HD domain in term of DNA binding and recognition. As demonstrated in the structural and functional study of PAX_PAIRED_-DNA complex, the RED sub-domain is not essential in DNA binding [23]. In line with this observation, many studies have shown that a single homeobox domain is not sufficient to mediate DNA binding/recognition for productive transactivation. Thus far, it has been reported that homeobox proteins often require: (1) the protein–protein interaction involving a second transcription factor; (2) inter-molecular dimerization; and (3) an extra intra-molecule DNA binding domain to bind DNA [24–27].

In DUX4, the presence of double homeobox appears to be sufficient for DNA recognition and transactivation. The gain of an extra HD domain in DUX4, possibly derived from gene reshuffle and infusion [19], gives rise to a powerful transcription factor for TAATCTAAT (DRE) site (Supplementary Figure 1). In B-ALL patients, nearly all the DUX4/IGHs contain a structural and functional intact double homeobox, leading to the hypothesis that the DUX4 double homeobox might play the central role in DNA binding and transactivation. Indeed, when the Reh cells were transfected with DUX4 fusion with different IGH tails, ERG_alt_ biogenesis were consistently observed (Fig. 1b, c). When the endogenous expression of DUX4/IGH was suppressed by shRNA, the target genes containing DRE sites were consistently downregulated (Supplementary Figure 6a). More importantly, when the DNA binding interface in either DUX4_HD1_ or DUX4_HD2_ was mutated, the binding affinity and transactivation function were severely impaired (Figs. 3 and 4). Altogether, these results suggest that the evolutionary tinkering of double homeobox design is an important mean to endow DUX4 with transactivation activity.

### Two-step clamp-like binding mechanism

In this report, the crystal structure of DUX4_HD2_-DRE reveals a novel two-step clamp-like DNA binding mechanism for double homeobox. In the asymmetric unit (ASU), it contains two HD2 molecules and a DNA duplex of TAATCTAAT (DRE) sequence. Based on homology modeling, a HD1 molecule can be mapped onto DRE site. Supportively, the C-termini of HD1 and the N-termini of HD2 are both correctly positioned, giving ample space to envisage the missing linker in between. More importantly, the QNR_HD1_ motif is predicted to insert into the DNA major groove engaging the direct hydrogen bonds with TAAT. In parallel, the RRRR motif of HD1 wraps around the DNA minor groove (Fig. 6). All these data suggest that: (1) HD1 can bind TAAT sequence in the same way as HD2 and (2) the presence of an extra HD domain might enhance its overall binding. Indeed, the BLI characterization showed that the double homeobox binds to a DRE sequence at a *K*_*D*_ value of 1.4 μM, >5 fold stronger than HD2 alone (Fig. 3a and Supplementary Figure 3a).

The two-step binding mode is further supported by structure-based mutagenesis. The single mutation of QNR_HD1_–QNR_HD2_ (i.e., N69A, N144A, R73A, R148A) could ~100% inhibit transactivation, suggesting both HD1 and HD2 are essential. In marked contrast, single mutations of RRRR_HD1_–RRKR_HD2_ were less dramatic, implying that (1) poly-Arg motif is less critical when compared to the QNR motif; (2) the existence of cooperative binding between HD1 and HD2. This is further supported by the observation that double mutations of RRRR_HD1_–RRKR_HD2_ (i.e., R20/95A, R21/96A, R22/K97A, R23/98A) resulted a complete loss of transactivation even in the presence of QNR_HD1_–QNR_HD2_ motifs (Fig. 4). This is also the case in BLI characterization, the poly-Glu mutation of RRRR–RRKR resulted in no binding between double homeobox and DNA (Supplementary Figure 3b). These data appear to suggest that the DNA binding of poly-Arg motifs might precede the interaction of TAAT-QNR. Based on these observations, we proposed a two-step clamp-like DRE recognition mechanism by DUX4 double homeobox (Fig. 6). In the first step, the highly positively charged RRRR_HD1_–RRKR_HD2_ is important to direct DUX4 towards its DNA substrate (via electrostatic interaction). In this step, the HD1 subdomain might take the leading role to make the first kiss with target DNA. The non-specific association between RRRR_HD1_–RRKR_HD2_ with DNA in turn enables the double homeobox to recognize the DRE site via QNR_HD1_–QNR_HD2_. The double kiss between TAATs and QNR motifs might be critical to “clamp” DUX4-DRE into an intimate association that enables productive transactivation of its target genes (Fig. 6). Similar DNA binding mode should also present in WT DUX4 (Supplementary Figure 5). In addition, the CTD domain, which clear contributes to DUX4/IGH-driven transactivation (Fig. 5), should remain an interesting subject for future investigation in the context of WT DUX4 and its oncogenic derivatives.

### Oncogenic potential of DUX4-DRE

It has been reported that homeobox-containing protein including PRD-type subclass is involved in hematopoiesis and leukemogenesis [28]. The abnormal expression of PAX5, a member of the PRD-type subclass, derived from chromosome translocation with the *IGH* locus can deregulate the lymphoid cell gene expression program and contribute to B and T-lineage neoplasms [29–31]. Recent studies showed that, the homeobox-containing DUX4 can also translocate into the *IGH* locus, and the aberrant expression of DUX4 can cause B-cell leukemia in immunocompromised mice [3]. A follow-up study by Zhang and co-workers showed that the aberrant expression of DUX4/IGHs were constantly associated with the production of a novel ERG_alt_ isoform that can also contribute to leukemogenesis *in vivo* [4]. These observations have led to the proposal of DUX4/IGH-ERG_alt_ axis in B-ALL development. However, not all patients in DUX4/IGH subgroup were ERG_alt_ positive (Supplementary Table 2), suggestive of other genetic lesion in B-ALL. The RNA-seq data mining of our 172 patients showed that the expression profile of DUX4/IGH positive ALL was significantly different from other B-ALL subtypes (Fig. 1 and Supplementary Figure 1). Using FACS and apoptotic assays, we have found that AGAP1, like DUX4/IGH and ERG_alt_, could contribute to B cells proliferation and differentiation. However, it should be noted, cytoplasm-bound AGAP1 is not a transcription factor like ERG or ERG_alt_, and its oncogenic pathway to B-cell arrest is not yet clear. Further investigations such as *in vivo* study in the background of *AGAP1* knock-out mice should be carried out to characterize its leukemogenic cross-talk with DUX4/IGH and ERG_alt_.

## Electronic supplementary material

Supplementary Text(DOC 71 kb)

Supplementary Table 1(DOC 34 kb)

Supplementary Table 2(DOCX 13 kb)

Supplementary Figure 1(PDF 847 kb)

Supplementary Figure 2(PDF 1197 kb)

Supplementary Figure 3(PDF 80 kb)

Supplementary Figure 4(PDF 487 kb)

Supplementary Figure 5(PDF 71 kb)

Supplementary Figure 6(PDF 228 kb)
