# Supplementary material for: Structural basis of DUX4/IGH-driven transactivation
Source: Leukemia. 2018 Mar 15;32(6):1466–76. doi: 10.1038/s41375-018-0093-1 (PMC5990521; doi:10.1038/s41375-018-0093-1)
Supplement: Supplementary file 1 — Supplementary Text(DOC 71 kb) [file 41375_2018_93_MOESM1_ESM.doc]

**Structural basis of DUX4/IGH-driven transactivation**

Xue Dong^*^, Weina Zhang^*^, Haiyan Wu^*^, Jinyan Huang^*^, Ming Zhang, Pengran Wang, Hao Zhang, Zhu Chen, Sai-Juan Chen^Ψ^, Guoyu Meng^Ψ^

State Key Laboratory of Medical Genomics, Shanghai Institute of Hematology, Rui-Jin Hospital, Shanghai JiaoTong University School of Medicine and School of Life Sciences and Biotechnology, Shanghai JiaoTong University, 197 Ruijin Er Road, Shanghai 200025, China; Key Laboratory of Systems Biomedicine, Shanghai Center for Systems Biomedicine, Shanghai JiaoTong University, 800 Dong Chuan Road, Shanghai 200240, China.

**Running title: Crystal structure of DUX4_HD2_-DRE**

^*^ equal contribution

^Ψ^co-senior and corresponding authors

E-mail: [guoyumeng@shsmu.edu.cn](mailto:guoyumeng@shsmu.edu.cn) and sjchen@stn.sh.cn

Tel: 0086 (0) 2164370045-610730

Fax: 0086 (0) 2164743206

**Keywords**: acute lymphoblastic leukemia, DUX4/IGH, DUX4-Responsive-Element, transactivation, ERG_alt_

**Materials and methods**

***Protein expression and purification***

The DNA fragments encoding residues 94-153 (DUX4_HD2L_) and 99-150 (DUX4_HD2S_) of human DUX4 protein were cloned into a bacterial expression vector pET15b under the T7 promoter, respectively. The expression vectors containing the target genes were transformed into *Escherichia coli* BL21 (DE3) cells for protein production. The recombinant proteins containing a N-terminal cleavable His-tag were induced with 500 mM IPTG (Sangon) until the optical density at 600 nm reached 0.6. Then the cells were grown at 16°C for 18 hours before harvest by centrifuge (4,000 rpm, 20 min).

The collected cells were resuspended in 20 mM Tris (pH 8.0), 100 mM NaCl and lysed using a cell cracker (JNBIO) with 20 kg/cm^2^ pressure. Cell debris was removed by centrifugation and the clear lysate was loaded onto a pre-equilibrated nickel sepharose column (HisTrap, GE Healthcare). The non-specific binding protein/comtaminants were washed off with 20 mM Tris (pH 8.0), 20 mM imidazole, 500 mM NaCl. The target proteins were eluted with 20 mM Tris (pH 8.0), 300 mM imidazole, 500 mM NaCl. The N-terminal His-tag was removed by recycling the eluate over a pre-equilibrated nickel column after thrombin digestion (4°C, >20 hours). DUX4_HD2L_ and DUX4_HD2S_ were purified further with a cation-exchange sepharose column (SP, GE Healthcare). Then target proteins were then subjected to a final polishing step by gel filtration (S100, GE Healthcare). The correct mass of the target protein was confirmed by MS analysis, and the purity of >95% was assessed by SDS-PAGE.

Synthetic single stranded DNA oligonucleotides of 5′-TTC**TAATCTAAT**CA-3′ and 5′-TG**ATTAGATTA**GAA-3′ (in which the DRE site is shown in bold and underscored) were re-suspended in sterile water, respectively. In order to obtain double stranded DNA, the oligonucleotides were mixed at a 1:1 molar ratio, then annealed under 95 °C for 10 min and slowly cooled to 4°C. The annealed DNA_DRE_ was concentrated to a final concentration of 80 mg/ml as judged by the absorbance at 260 nm and then stored under -80°C for further use.

***Crystallization, cryoprotection and data collection***

All crystallization experiments were carried out using the hanging drop vapor-diffusion method. Crystal of the DUX4_HD2S_ (36 mg/ml) was obtained in a reservoir solution containing 30% PEG4000 and 120 mM ammonium sulfate within 2 weeks at 4 °C. For co-crystallization of the DUX4_HD2L_-DNA_DRE_, the double-stranded DNA_DRE_ and DUX4_HD2L_ protein (35 mg/ml) were incubated at a 1:1 molar ratio at 4 °C for 30 min. The mixture was then subjected to crystallization screen at 20°C. The final crystallization condition of DUX4_HD2L_-DNA_DRE_ was 100 mM Hepes (pH 7.5), 18% PEG8000, 2% 2-propanol, 100 mM sodium acetate.

The crystals were flash-cooled in liquid nitrogen using 20% PEG400 as cryoprotectant. The diffraction data were collected in beamline station BL17U at Shanghai Synchrotron Radiation Facility (SSRF, Shanghai, China). Crystals of DUX4_HD2S_ (also known as *Apo*-DUX4_HD2_) diffracted to 1.5 Å and were in space group *P*2_1_2_1_2_1_ with cell dimensions of a = 25.2 Å, b = 25.4 Å and c = 72.6 Å. The DUX4_HD2L_-DNA_DRE_ (also known as DUX4_HD2_-DNA_DRE_) crystals diffracted to 2.6 Å and were in space group *P*4_1_2_1_2. The unit cell parameters were a = 51.6 Å, b = 51.6 Å, c = 166.6 Å. The diffraction data were processed, integrated and scaled using MOSFLM/SCALA^33^. The statistics of the data collection are shown in Supplementary Table 1.

***Phasing and structure refinement***

The initial phases of *Apo*-DUX4_HD2_ were determined by molecular replacement using aristaless homeodomain (PDB ID: 3A02) as search template. The autotracing program ARP/wARP1 was then used to produce an σ_A_-weighted 2Fo-Fc map for further manual model building. REFMAC5^34^ and PHENIX.REFINE^35^ were used for structural refinement. Intermittent manual building implemented in COOT^33^ was used to correct and improve the initial model produced by ARP/warp^36^. The *B*-factors were refined with TLS corrections^34^. For the complex structure, the refined coordinates of *Apo*-DUX4_HD2_ were used to estimate the initial phases. A similar model building process, combined with intermittent structural refinement using REFMAC5 and PHENIX.REFINE, was used to refine the complex of DUX4_HD2_-DNA_DRE_. The final model of *Apo*-DUX4_HD2_ contains 50 residues and 108 water molecules. Ramachandran statistics of *Apo*-DUX4_HD2_ calculated by PROCHECK^37^ showed that 100% of the atoms are in the most favored regions. As for DUX4_HD2_-DNA_DRE_, the final model contains 113 protein residues and 26 DNA nucleotides, of which 90.8, 6.4, 2.8% of the atoms are in the most favored, allowed and outlier regions, respectively. The detailed structure refinement statistics are reported in Supplementary Table 1. The coordinates of DUX4_HD2_ with or without DNA_DRE_ will be deposited into the Protein Database Bank.

***Biolayer interferometry (BLI) assay***

The binding affinities between recombinant DUX4_HD2_ (residues 94-153) or DUX4_HD1-2_ (residues 19-153) and DNA_DRE_ were measured using biolayer interferometry (BLI) technique implemented in an Octet Red 96 instrument (ForteBio). The 96-well black microplates were used. The biotin-DNA_DRE_ was immobilized on the SA biosensors (ForteBio). Each biosensor was then plunged into a well containing 200 μL of purified DUX4_HD2_ or mutants at a step-wise increase of concentrations from 0.5 to 32 μM. All experiments were done with reference wells (i.e. no protein). The association/dissociation curves of each concentration were obtained by the subtraction of control, with DNA_DRE_ immobilized on the probe. The dissociation constants (*K_D_*) were obtained by fitting saturation data with a nonlinear least-squares regression in Prism (GraphPad). RU max is the BLI signal at maximal binding in nanometers.

***Plasmids,*** ***cell culture and viruses packaging***

The cDNAs of DUX4/IGHs were amplified from B-ALL patients, and engineered into LEGO-iG2 vector or MigR1-IRES-GFP vector. HA-tag was fused in frame with DUX4/IGH for detection purpose (Abcam, ab9110). The DUX4/IGH mutants were obtained by site-directed mutagenesis. The shRNA sequences engineered in the PLVX-shRNA2 vector were designed as follows:

5’-ACCCUGUGUGUCUCAGUUCAUA-3’ (sh-DUX4/IGH),

5’- GUGCACAUCAGCCAGACAAGU-3’ (sh-AGAP1-1),

5’- GACCUAUCAUCCCAGUUUACA-3’ (sh-AGAP1-2),

5’-GAGAAGGAACCAACUGAAGAU-3’ (sh-STAP1-1),

5’- GCCUUACUGAGCAGAAUUCAA-3’ (sh-STAP1-2),

5’-GCACAGUCCUCACUUUCAUCU-3’ (sh-LHFPL2-1),

5’- GCCUCUAGUGACAAAGUACAGGAAGA-3’ (sh-LHFPL2-2),

5’-GGCAUUCACGCACUACAACAU-3’ (sh-MPPED2-1),

5’- GGGAAUCAUGAACUGACAUUU-3’ (sh-MPPED2-2),

5’-GCUAUGGAGGUCAUCUGCAAUAGUA-3’ (sh-CHST2),

5’-GUGAAGAGCUCCAGAGAAAUA-3’ (sh-CLEC12A-1),

5’- GGUAUGAGAGUGGAUAAUA-3’ (sh-CLEC12A-2).

The 293T cells were cultured in DMEM medium supplemented with 10% FBS. Reh cells and NALM6 cells were cultured in RPMI 1640 medium supplemented with 10% FBS. These cells are cultured in Shanghai Institute of Hematology, and tested routinely for mycoplasma contamination. The lentiviruses for Reh cells were prepared by transfection of 293T cells with WT or mutant DUX4/IGH-LEGO-iG2, pMD2.G, psPAX2 and RSV using Lipofectamin 2000 (Invitrogen). Retroviruses harboring various DUX4/IGHs for NALM6 cells were prepared by a transient transfection of 293T cells with WT or mutant DUX4/IGH-MigR1, VSVG and gagpol. The lentiviruses for shRNA-mediated knock-down experiments were obtained using 293T cells co-transfected with PLVX-shRNA2, pMD2.G and psPAX2.

***Transactivation of ERG_alt_ by DUX4/IGH***

The Reh or NALM6 cells containing DUX4/IGH or mutants (i.e. structure-based mutants and CTD truncated mutants) were lysed with SDS buffer and sonication on the fourth day after transduction. For western blot, the clear lysate was resolved on a 12% SDS-PAGE gel and transferred onto a PVDF membrane. The transactivation of ERG_alt_ was detected by antibody against human ERG protein (Abcam, ab92513).

In parallel, the real-time PCR technique was used to confirm the DUX4-driven tansactivation at mRNA level. The total mRNA was extracted from Reh or NALM6 cells on the fourth day after transduction. Isolated RNA was reversetranscribed into cDNA using random primers and MMLV (Invitrogen). The expression of DUX4 target genes was detected using quantitative real-time PCR, which was performed using ABIPRISM 7500 (Applied Biosystems) with QuantiNova SYBR Green PCR Kit (Qiagen). GAPDH was used as an internal control.

***Luciferase assay***

The DNA binding region of *AGAP1* was cloned into the pGL4.15 firefly luciferase reporter vector (Promega). The reporter construct, a Renilla luciferase vector used for control of transfection efficiency, and WT DUX4/IGH or its mutant plasmids were cotransfected into 293T cells using Lipofectamin 2000. Twenty-four hours after transfection, cells were harvested for determination of luciferase activities using the Dual-luciferase reporter assay kit (Promega).

***B cell differentiation assay***

Mouse bone marrow lineage negative (Lin-) cells were isolated using a lineage depletion kit (MiltenyiBiotec), and then c-Kit^Low^ cells were sorted by flow cytometry technique using FACSArialII (BD). Lin- and c-Kit^Low^ cells were transduced with various retroviruses containing *DUX4/IGH* and mutants. The transfected cells were cultured for 5 days on an OP9 monolayer in IMDM containing 20% FBS, SCF (20 ng/ml), Flt3L (50 ng/ml) and IL-7 (50 ng/ml). The effects on lymphoid lineage differentiation by *DUX4/IGH* and its mutants as well as its target genes were assessed by flow cytometry using antibodies against mouse CD19 and Mac-1.

***Gene-expression analysis***

The raw unfiltered RNA-seq reads were aligned to human reference genome hg19 using Hisat2 (version 2.0.5), with default parameters. To ensure the highest possible level of data quality, samples with reads number less than 30 megabyte were not included in the analysis. The transcription expression levels were calculated as FPKM values as previously described^38^. Differentially expressed genes in patients with DUX4 fusions and other groups were computed using DESeq package.

***Patients and samples***

Adult patients were enrolled in a Shanghai Institute Hematology protocol (Chinese Clinical Trial Registry, number ChiCTR-RNC-14004969, for sample collection, and ChiCTR-ONRC-14004968, for treatment). Pediatric patients were mostly enrolled in the Shanghai Children's Medical Center ALL-2005 protocol (Chinese Clinical Trial Registry, number ChiCTR-ONC-14005003).

***Structure-based characterization of WT DUX4***

WT DUX4 was amplified from human genomic DNA and cloned into MigR1-IRES-GFP vector. Structure-based mutants as described above were transfected into 293T cells. After 24 hours, 293T cells were digested and subjected to apoptotic analysis. The apoptotic cells were labeled with Annexin-V and detected by flow cytometry. As for luciferase assay in the context of WT DUX4, the DNA binding region of *ERG_alt_* was cloned into the pGL4.15 firefly luciferase reporter vector and the luciferase activities of WT DUX4 and mutants were detected as described above.

***Statistical analysis***

All data was presents as mean ± SE, and statistical signficance was assessed through unparied two-tailed Students’ t-test using GraphPad Prism (*, P<0.05; **, P<0.01; ***, P<0.001).

**Figure legends**

**Supplementary Figure 1. DUX4-Responsive-Element (DRE).** a) The expression levels (FPKM) of *DUX4/IGH* and its target genes determined by RNA-seq, in B-ALL subgroups/patients. FPKM stands for Fragments Per Kilobase of transcript per Million mapped reads. The sub-groups are highlighted using the same color scheme as Figure 1a. b) Re-analysis of published ChIP-seq data identified the DNA binding sites of DUX4. The DRE sites are highlighted with boxes. c) The consensus DUX4 binding sites derived from ChIP-seq analysis.

**Supplementary Figure 2**. **The residues and surfaces governing the formation of DUX4_HD2_-DNA_DRE_.** a) The N-terminal R_95_RKR_98_ loop is highly flexible and might adopt different configurations for the binding of DNA minor groove. The two HD2 molecules in ASU are superimposed in the context of DNA binding. Two sets of RRKR residues, showed in stick representation, are colored in red and green, respectively. Upon DNA binding, Arg96/Lys97 (green) or Arg95/Lys97 (red) are mobilized to make direct contract with the phosphate groups of DNA in the minor groove. b) Structural superimposition between *Apo*- and DNA-bound structures (red and green, respectively). The residues engaging in the interaction in DNA major groove are shown in stick representations. c) Sequence alignment of double homeobox domains in DUX family proteins. The residues of N-terminal RRRR_HD1_-RRKR_HD2_ loops and the code-reading QNR_HD1_-QNR_HD2_ residues in the α3 helices are colored in green and red, respectively. The HD2 sequence characterized by X-ray crystallography is boxed. The residues delineating the central positively charged pocket is underscored. d) Sequence alignment between DUX4_HD2_ and other homeobox proteins. The DNA binding sites are highlighted with the same color scheme described above. The invariant and highly conserved residues are showed with “*” and “:”, respectively. The secondary structures of α1, α2, α3 helices of DUX4_HD2_ are shown with cylinder above the sequences. Similar color/coding scheme is used to highlight poly-Arg/QNR motifs and other DNA binding residues in DUX4_HD2_.

**Supplementary Figure 3. The BLI characterization of double homeobox domain.** a) The interaction between WT HD1-HD2 and DNA_DRE_. b) The poly-Glu mutation in DNA binding interface. A gradient concentration of 0.25 to 16 μM purified HD1-HD2 were used. The DNA substrate was immobilized. Left: The experimental titration of double homeobox. Right: The *K_D_* values derived from Scatchard plots.

**Supplementary Figure 4**. **DUX4/IGH-driven transactivation in NALM6 cells.** Western blot analysis of ERG_alt_ production in NALM6 cells expressing WT DUX4/IGH or mutants. a) Perturbation of poly-Arg motif in HD1. b) Perturbation of QNR motif in HD1. c) Perturbation of poly-Arg motif in HD2. d) Perturbation of QNR motif in HD2. e) Double mutation in poly-Arg motifs of HD1-HD2. f) Double mutation in QNR motifs of HD1-HD2. All experiments have been done at least with three independent replicates.

**Supplementary Figure 5.** **WT DUX4 shares similar DNA binding mode as DUX4/IGH.** a) Impact of structure-based mutations in the context of WT DUX4 in 293T cells. After overexpressing WT DUX4 and mutants in 293T cells, apoptotic cells were labeled with Annexin-V and detected by flow cytometry. b) Luciferase assay of WT DUX4 upon ERG_alt_ binding site. The mutations in QNR motifs impaired the transactivation of ERG_alt_ by WT DUX4. ***, p < 0.001.

**Supplementary Figure 6. Functional characterization of DUX4/IGH target genes.** a) The relative expression of DUX4/IGH target genes in NALM6 cells transduced with lentiviruses encoding DUX4 shRNA or scramble shRNA as control. b) Effect of *DUX4/IGH*, *AGAP1* and other DUX4/IGH target genes on B cell differentiation *ex vivo*. Top panel, statistics analysis of *ex vivo* assays. **, p < 0.01. ***, p < 0.001. Bottom panel, FACS plots of each DUX4/IGH target genes. c) Apoptotic effect of knock-down of DUX4/IGH target genes by shRNA in NALM6 cells. The apoptotic cells were labeled with Annexin-V and detected by FACS. Top panel, the apoptotic effect. *, p < 0.05. **, p < 0.01. Bottom panel, the knock-down efficiency of shRNA clones of indicated genes. d) Luciferase assay of DUX4/IGH-driven *AGAP1* transactivation. RRKR_HD2_/poly-A, poly-Ala mutation of RRKR motif in HD2. QNR_HD2_/poly-A, poly-Ala mutation of QNR motif in HD2*.* The DUX4/IGH-binding site of *AGAP1*, revealed by ChIP analysis (Supplementary Figure 1), was used in this study.

**Supplementary Table 1. Data collection and structure refinement statistics of *Apo*-DUX4_HD2_ and DUX4_HD2_-DNA_DRE_.**

**Supplementary Table 2. The details of DUX4/IGH patients.**

**REFERENCEs**

33. CCP4. The CCP4 suite: programs for protein crystallography. *Acta Crystallogr D Biol Crystallogr* 1994 Sep 01; **50**(Pt 5)**:** 760-763.

34. Winn MD, Isupov MN, Murshudov GN. Use of TLS parameters to model anisotropic displacements in macromolecular refinement. *Acta Crystallogr D Biol Crystallogr* 2001 Jan; **57**(Pt 1)**:** 122-133.

35. Adams PD, Afonine PV, Bunkoczi G, Chen VB, Davis IW, Echols N*, et al.* PHENIX: a comprehensive Python-based system for macromolecular structure solution. *Acta Crystallogr D Biol Crystallogr* 2010 Feb; **66**(Pt 2)**:** 213-221.

36. Perrakis A, Morris R, Lamzin VS. Automated protein model building combined with iterative structure refinement. *Nat Struct Biol* 1999 May; **6**(5)**:** 458-463.

37. Laskowski RA, MacArthur MW, Moss DS, Thornton JM. PROCHECK: a program to check the stereochemical quality of protein structures. *Journal of Applied Crystallography* 1993; **26**(2)**:** 283-291.

38. Anders S, Pyl PT, Huber W. HTSeq--a Python framework to work with high-throughput sequencing data. *Bioinformatics* 2015 Jan 15; **31**(2)**:** 166-169.
