# Supplementary material for: Structural basis of DUX4/IGH-driven transactivation
Source: Leukemia. 2018 Mar 15;32(6):1466–76. doi: 10.1038/s41375-018-0093-1 (PMC5990521; doi:10.1038/s41375-018-0093-1)
Supplement: Supplementary file 2 — Supplementary Table 1(DOC 34 kb) [file 41375_2018_93_MOESM2_ESM.doc]

**Supplementary Table 1. Data collection and structure refinement statistics of *Apo*-DUX4_HD2_ and DUX4_HD2_-DNA_DRE_*.***

**Data collection**

Protein *Apo*-DUX4_HD2_ DUX4_HD2_-DNA_DRE_ SafDAA A S

Space group *P*2_1_2_1_2_1_ *P*4_1_2_1_2

Unit cell dimension (Å)

a 25.2 51.6

b 25.4 51.6

c 72.6 166.6

Molecule per ASU 1 2

Derivative Native Native

Source/Station^a^ BL17U BL17U

Wavelength (Å) 0.9792 0.9791

Resolution range (Å) 36.3 - 1.50 83.3 - 2.62

Observations (*I*/σ(*I*) > 0) 47019 69864

Unique reflections (*I*/σ(*I*) > 0) 7483 (767) 7363 (1026)

High resolution shell (Å) 1.58 - 1.50 2.76 - 2.62

*R*_sym_ (%)^b,c^ : 7.0 (8.9) 9.9 (155.7)

<*I*/σ(*I*)>^c^: 19.2 (15.6) 11.5 (1.5)

Completeness^c^ (%): 94.6 (100.0) 99.9 (100.0)

Redundancy^c^: 6.3 (6.7) 9.5 (9.9)

**Structure refinement**

Resolution range (Å) 24.0 - 1.5 83.3 - 2.62

*R*-factor (%) 18.9 27.3

*R*-factor (high resolution shell)^d^ 17.4 43.7

*R*_free_ (%)^e^ 21.5 29.9

*R*_free_ (high resolution shell) 23.1 50.3

Total number of non-hydrogen atoms 515 1458

Protein atoms 407 931

DNA atoms 0 527

Water molecules 108 0

R.m.s. deviations:^f^

Bond length (Å) 0.010 0.005

Bond angle (º) 1.13 0.830

Wilson *B*-factor (Å^2^) 5.6 72.9

Average *B*-factor protein atoms (Å^2^) 9.7 77.2

Ramachandran statistics (%)

Most favored region 100 90.8

Allowed regions 0 6.4

Outlier regions 0 2.8

^a^Beamline designations refer to the Shanghai Synchrotron Radiation Facility, Shanghai, P. R. of China. ^b^*R*_sym_=Σ(*I*-<*I*>)2/Σ*I*^2^. ^c^overall, high resolution shell in parentheses. ^d^high resolution shell: 1.554 - 1.500 Å (*Apo*-DUX4_HD2_) and 2.717 - 2.622 Å (DUX4_HD2_-DNA_DRE_). ^e^*R*_free_ calculated using 5% of total reflections omitted from refinement. ^f^R.m.s. deviations report root mean square deviations from ideal bond lengths/angles^39^.

39. Engh RA, Huber R. Accurate bond and angle parameters for x-ray protein structure refinement. Acta Crystallogr. 1991;A47:392-400.
