# Supplementary material for: Structural basis of DUX4/IGH-driven transactivation
Source: Leukemia. 2018 Mar 15;32(6):1466–76. doi: 10.1038/s41375-018-0093-1 (PMC5990521; doi:10.1038/s41375-018-0093-1)
Supplement: Supplementary file 3 — Supplementary Table 2(DOCX 13 kb) [file 41375_2018_93_MOESM3_ESM.docx]

**Supplementary Table 2. The details of DUX4-IGH patients.**

| **Patient ID** | **Fusion gene** | **Age (yrs)** | **Gender** | **WBC**  **(×109/L)** | **CR** | **Death** | **Relapse** | **ERG_alt_** |
| --- | --- | --- | --- | --- | --- | --- | --- | --- |
| S1 | DUX4-IGH | 8.9 | Female | 3.4 | 1 | 0 | 0 | 1 |
| S2 | DUX4-IGH | 46 | Female | 3.3 | 1 | 0 | 0 | 1 |
| S3 | DUX4-IGH | 18 | Female | 103.8 | 1 | 0 | 0 | 1 |
| S4 | DUX4-IGH | 18 | Female | 18.7 | 1 | 1 | 1 | 1 |
| S5* | DUX4-IGH | 2.9 | Male | 27.2 | 1 | 0 | 0 | 0 |
| S6 | DUX4-IGH | 19 | Female | 18.9 | 1 | 1 | 1 | 1 |
| S7 | DUX4-? | 18 | Male | 4 | 1 | 0 | 0 | 1 |
| S8 | DUX4-IGH | 18 | Male | 76.5 | 1 | 0 | 0 | 1 |
| S9* | DUX4-IGH | 9.2 | Male | 55.6 | 1 | 0 | 1 | 0 |
| S10 | DUX4-IGH | 11.1 | Male | 1.9 | 1 | 0 | 0 | 1 |
| S11 | DUX4-? | 5.9 | Female | 5.7 | 1 | 0 | 0 | 1 |

*** The patients, who did not show ERGalt expression as determined by RNA-seq.**
