# Supplementary figures and images for: Structural basis of DUX4/IGH-driven transactivation
Source: Leukemia. 2018 Mar 15;32(6):1466–76. doi: 10.1038/s41375-018-0093-1 (PMC5990521; doi:10.1038/s41375-018-0093-1)

Supplementary Figure 1 Dong et al, 2018

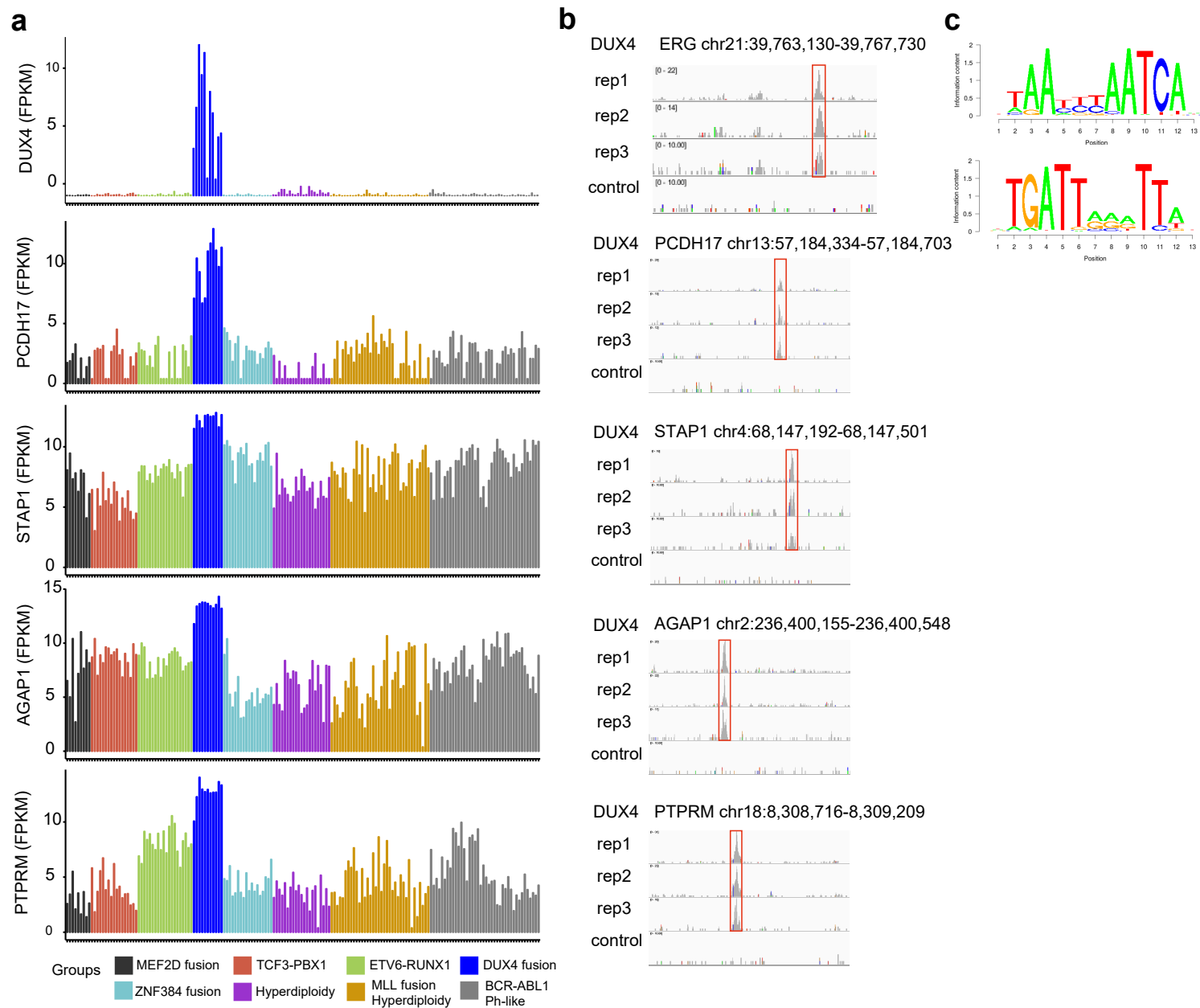

Supplement: Supplementary file 4 — Supplementary Figure 1(PDF 847 kb) [file 41375_2018_93_MOESM4_ESM.pdf]

Supplementary Figure 2 Dong et al, 2018

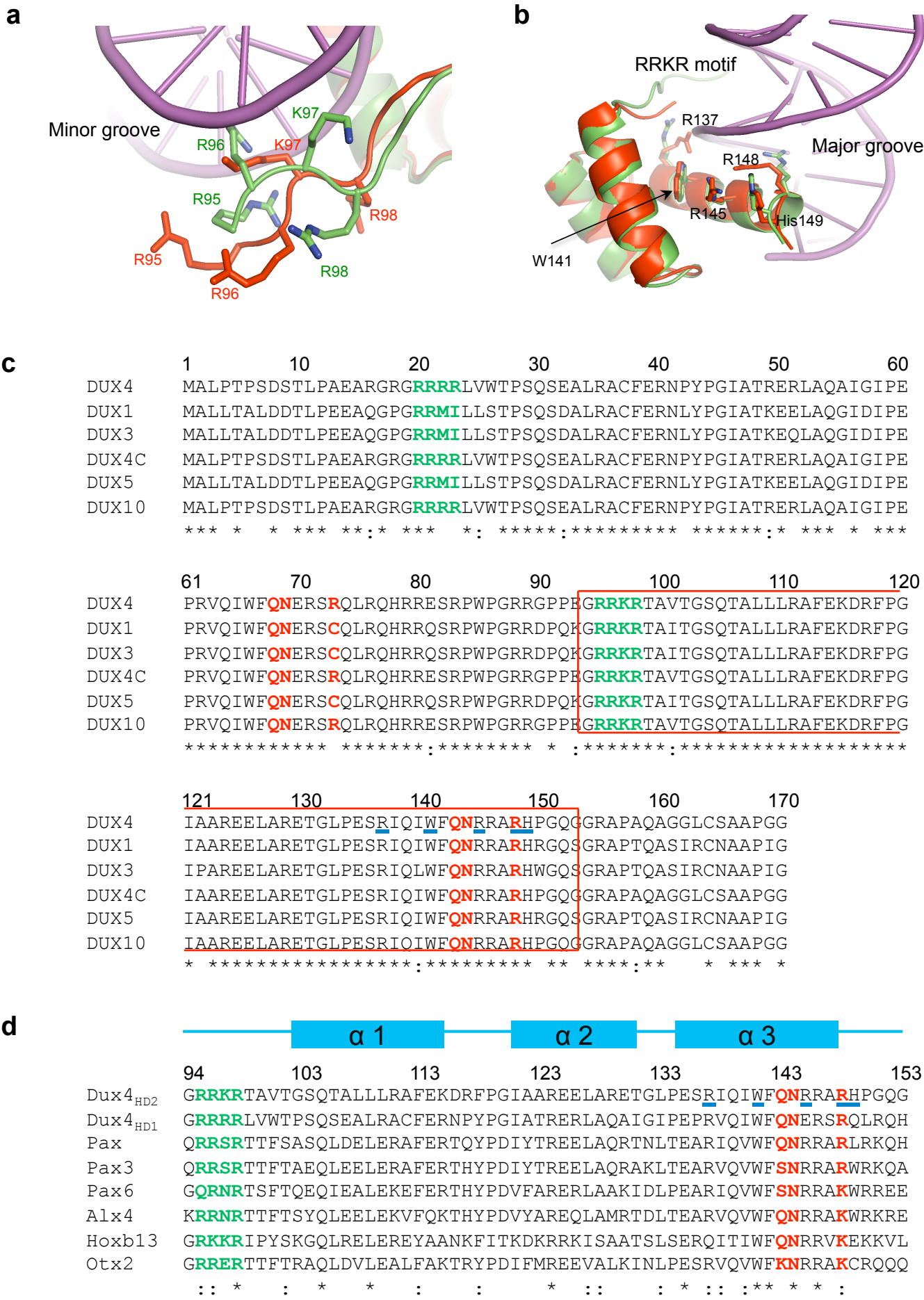

Supplement: Supplementary file 5 — Supplementary Figure 2(PDF 1197 kb) [file 41375_2018_93_MOESM5_ESM.pdf]

Supplementary Figure 3 Dong et al, 2018

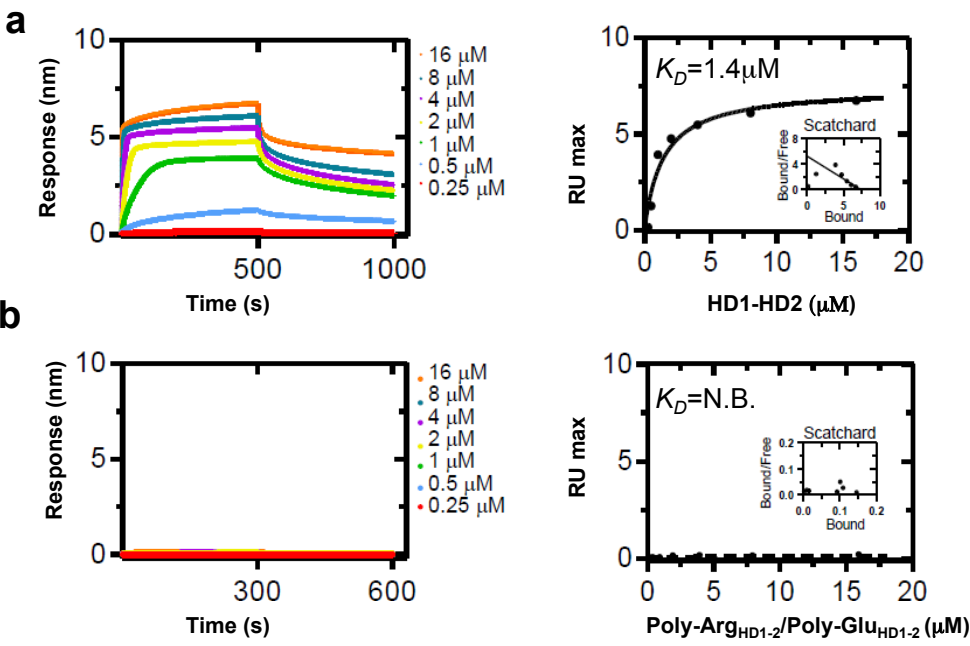

Supplement: Supplementary file 6 — Supplementary Figure 3(PDF 80 kb) [file 41375_2018_93_MOESM6_ESM.pdf]

Supplementary Figure 4 Dong et al, 2018

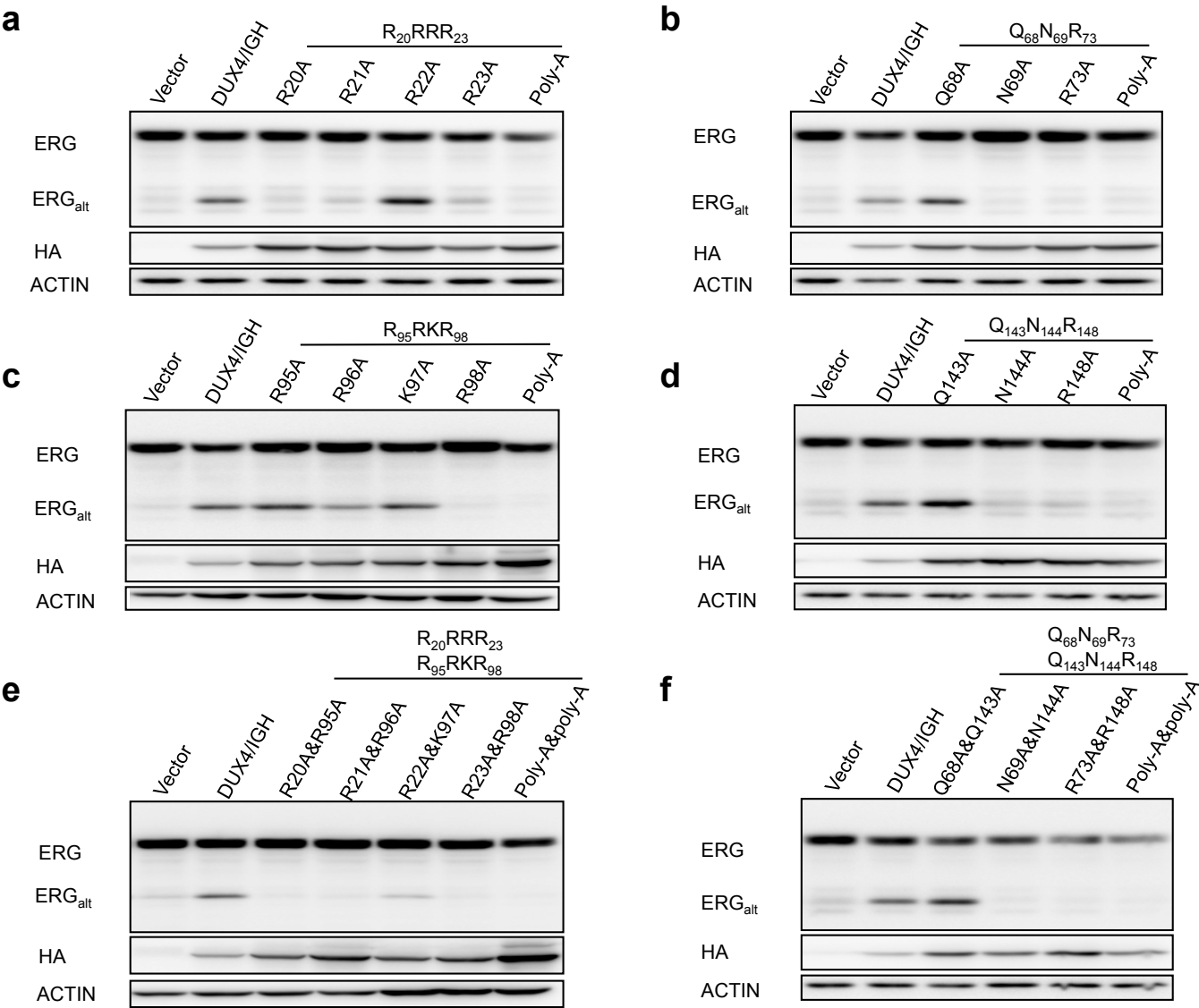

Supplement: Supplementary file 7 — Supplementary Figure 4(PDF 487 kb) [file 41375_2018_93_MOESM7_ESM.pdf]

# Supplementary Figure 5 Dong et al, 2018

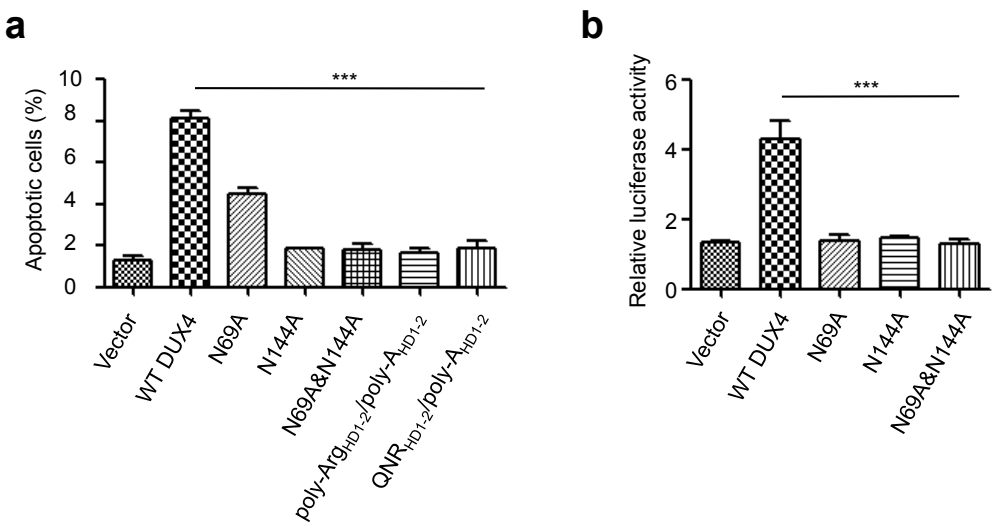

Supplement: Supplementary file 8 — Supplementary Figure 5(PDF 71 kb) [file 41375_2018_93_MOESM8_ESM.pdf]

Supplementary Figure 6 Dong et al, 2018

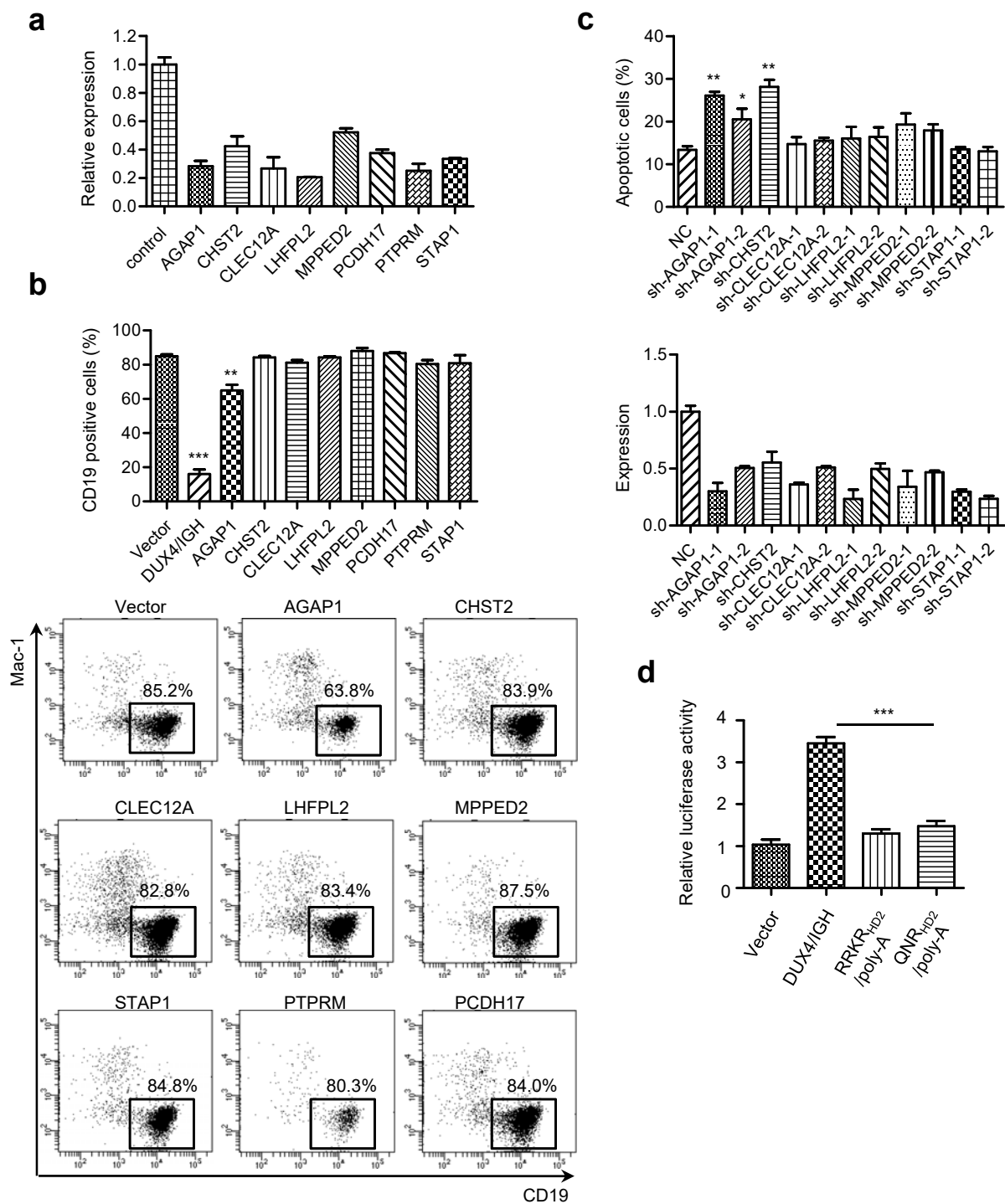

Supplement: Supplementary file 9 — Supplementary Figure 6(PDF 228 kb) [file 41375_2018_93_MOESM9_ESM.pdf]
